# Enhanced hydrogen evolution reaction activity through samarium-doped nickel phosphide (Ni_2_P) electrocatalyst

**DOI:** 10.1038/s41598-024-66775-7

**Published:** 2024-07-22

**Authors:** Ali Shahroudi, Sajjad Habibzadeh

**Affiliations:** Surface Reaction and Advanced Energy Materials Laboratory, Chemical Engineering Department, Amirkabir University of Technology (Tehran Polytechnic), Tehran, Iran

**Keywords:** Electrocatalyst, Hydrogen evolution reaction, Nickel phosphide, Samarium, Doping, Electrochemistry, Energy, Green chemistry, Materials chemistry, Surface chemistry, Chemical synthesis

## Abstract

Hydrogen evolution reaction (HER) stands out among conventional hydrogen production processes by featuring excellent advantages. However, the uncompetitive production cost due to the low energy efficiency has hindered its development, necessitating the introduction of cost-effective electrocatalysts. In this study, we introduced samarium doping as a high-potential approach to improve the electrocatalytic properties of nickel phosphide (Ni_2_P) for efficient HER. Samarium-doped Ni_2_P was synthesized via a facile two-step vapor–solid reaction technique. Different physical and electrochemical analyses showed that samarium doping significantly improved pure Ni_2_P characteristics, such as particle size, specific surface area, electrochemical hydrogen adsorption, intrinsic activity, electrochemical active surface area, and charge transfer ability in favor of HER. Namely, Ni_2_P doped with 3%mol of samarium (Sm_0.03_Ni_2_P) with a Tafel slope of 67.8 mV/dec. and overpotential of 130.6 mV at a current density of 10 mA/cm^2^ in 1.0 M KOH solution exhibited a notable performance, suggesting Sm_0.03_Ni_2_P and samarium doping as a remarkable electrocatalyst and promising promoter for efficient HER process, respectively.

## Introduction

Ever-increasing world energy demand coupled with the distressing environmental issues posed by fossil fuel overconsumption has necessitated the urgent development of eco-friendly and sustainable energy sources^1^. In this regard, hydrogen has emerged as a promising alternative, considering its great energy density and clean combustion^2^. Among different hydrogen production methods, hydrogen evolution reaction (HER) excels in many aspects by producing high-purity hydrogen from abundant water resources at moderate conditions without undesired emissions^3^. However, the comparatively high HER-driven hydrogen cost due to poor energy efficiency has hindered its reasonable development and resulted in a small share of the hydrogen market, emphasizing the employment of active electrocatalysts^4^. Pt-group metals are known as the most active HER electrocatalysts. However, their scarcity and corresponding expensiveness have restricted their practical usage, highlighting the introduction of active yet affordable materials to enable the deserved development of HER^5^.

Transition metal-based materials with low price, high abundance, and relatively easy preparation have shown notable performance in HER among which nickel phosphides, and particularly the Ni_2_P crystalline phase by showing outstanding performance are regarded as future HER electrocatalysts^6^. Many researchers have considered the performance of pristine Ni_2_P in HER^7–10^. However, despite promising activity, its performance has not yet been suited to real applications and requires further improvement. Thus, several approaches have been implemented, among which doping has shown significant potential. Thus far, transition metals or heteroatoms have mainly been studied as doping agents in HER electrocatalysis^11–20^. However, despite their excellent capability, rare-earth (RE) elements have not been well considered, and the HER electrocatalysis research area lacks sufficient studies on this area.

RE elements are 17 elements comprising scandium, yttrium, and 15 elements of the lanthanide series^21^. Unlike their title, RE elements are abundant in the Earth's crust^22^. However, since they have been widely dispersed over the Earth and are not concentrated in one location, they have historically been classified as *rare-earth* elements^23^. RE elements feature unique chemical, electronic, magnetic, and optical properties that have been used in a wide variety of applications. Their 4f orbitals allow various energy level transitions, making RE elements ideal for electrochemical applications such as supercapacitors, batteries, and electrocatalysis^24^. Namely, Engel-Brewer theories predict that doping RE-elements with empty or half-filled d-subshell orbitals, like cerium and samarium, into transition metals with available unpaired d-subshell orbitals, such as cobalt and nickel, can have a synergistic effect in favor of HER. This phenomenon has been illustrated by several theoretical computational studies. Moreover, multiple experimental researches from an empirical perspective have demonstrated the great potential of RE elements in HER^25–30^.

In a pioneering study, Morse and Greene^31^ explored the electrocatalytic activity of thirteen RE elements in HER and reported noteworthy performance for them. However, the scientific community shifted its focus towards combining RE elements with transition metals, considering their relatively greater capability^25,32^. In this regard, Miles^33^ applied LaNi_5_ alloy for the first time in HER, opening doors for researchers to investigate this alloy further^34^. Subsequently, various alloys consisting of transition metals combined with different RE elements were also considered^35–37^, leading to the gradual development of research in this area. However, in the early studies, casting methods were predominantly employed to alloy transition metals and RE elements, which were deemed less desirable for several reasons. Therefore, researchers have increasingly used more advanced methods like doping to involve RE elements in recent years.

Gao et al.^26^ studied cerium-doped CoP electrocatalyst in HER and demonstrated the notable effect of cerium doping in enhancing the performance of CoP. This finding was also reported in several similar studies^27–30^. Subsequently, researchers started to investigate the effect of cerium doping on nickel phosphides^38–40^. In a comprehensive work, our research group conducted a study on cerium-doped nickel phosphide (Ni_2_P) using computational and experimental approaches, where the significant impact of cerium on the performance of Ni_2_P was revealed^41^. The promising results of this study, along with the similar electronic structure of Lanthanides, motivated us to investigate further the effects of other RE elements to broaden the horizons in this field of research. Therefore, we focused on samarium doping in the present study to achieve efficient HER electrocatalysis.

This study introduces samarium as a novel promoter to enhance the electrocatalytic performance of nickel phosphide (Ni_2_P) in HER. Samarium-doped Ni_2_P is prepared via a two-step vapor–solid reaction method. The effects of samarium doping are comprehensively analyzed from various aspects by performing several physical and electrochemical characterization tests. Concisely, samarium doping remarkably improves the electrocatalytic properties of pure Ni_2_P, suggesting samarium as a remarkable dopant for efficient HER.

## Experimental methods

### Materials

NiSO_4_.6H_2_O, NaH_2_PO_2_.H_2_O, Sm(NO_3_)_3_.6H_2_O and NaOH (precursors with high purity were obtained from Sigma-Aldrich company and consumed as received.)

### Synthesis of the developed electrocatalysts

In this study, samarium doping was performed at 3 percentages, including 1, 3, and 5%mol, and the corresponding samples were named Sm_0.01_Ni_2_P, Sm_0.03_Ni_2_P, and Sm_0.05_Ni_2_P, respectively. Sm-doped Ni_2_P was synthesized by a two-step vapor–solid reaction (VSR) process. Sm-doped Ni(OH)_2_ was initially prepared using a precipitation technique. Typically, 2.037 g of NiSO_4_.6H_2_O and 0.101 g of Sm(NO_3_)_3_.6H_2_O were dissolved in 155 ml of distilled water to make a precursor solution. Next, 15 ml of 1.0 M NaOH solution, as the precipitant, was added gradually to the precursor solution. The resultant mixture was moved into a 230 ml Teflon-lined stainless steel autoclave and was maintained for 12 h at 110 °C. Then, Sm-doped Ni(OH)_2_ precipitate was achieved, washed several times with distilled water, and dried naturally in air to result in Sm-doped Ni(OH)_2_ powder. In the second step, Sm-doped Ni_2_P was synthesized by phosphorizing Sm-doped Ni(OH)_2_ using a VSR. Typically, Sm-doped Ni(OH)_2_ powder and NaH_2_PO_2_.H_2_O with a molar ratio of 1:5 were heated in a furnace for 2 h at 350 °C. When the furnace reached room temperature, Sm-doped Ni_2_P was collected. Pure Ni_2_P was synthesized using a similar procedure without using the samarium precursor in the first step.

### Preparation of electrodes

A titanium sheet measuring 20 mm × 10 mm × 1 mm was used as the substrate. Prior to electrode preparation, the substrate was cleaned by washing with water, polishing with sandpaper, and etching with a 6.0 M HCl solution to remove impurities from the surface. 10 mg of the electrocatalyst was added to the mixture consisting of 200 µl of distilled water, 750 µl of ethanol, and 50 µl of a 5% wt polyvinylidene fluoride (PVDF) binder solution in N-Methylpyrrolidone (NMP). The mixture was then sonicated for 30 min to ensure a homogeneous ink. The electrocatalyst ink was subsequently dropped onto the prepared Ti sheet and left to dry at room temperature.

### Electrocatalyst characterization

X-ray diffraction (XRD) test was carried out to investigate the crystalline structure of electrocatalysts using an Inel EQUINOX 3000 X-ray diffractometer with Cu Kα radiation. Field emission scanning electron microscopy (FE-SEM) images were taken to consider the morphology of electrocatalysts by using a TESCAN Mira3 microscope equipped with an energy-dispersive X-ray spectrometer (EDS). X-ray photoelectron spectroscopy (XPS) was performed to analyze the surface chemical composition of samples using a Bestec EA10 plus spectrometer with aluminum radiation. The gas (nitrogen) sorption test was conducted to calculate electrocatalysts' specific surface area (SSA) using a Belsorp mini II surface area and pore size analyzer.

### Electrochemical measurements

Electrochemical measurements were performed using an AUTOLAB potentiostat in a typical three-electrode cell. The synthesized electrocatalyst, Ag/AgCl electrode, and graphite rod were used as the working, reference, and counter electrodes, respectively. All electrochemical measurements were carried out in 1.0 M KOH solution. The activity of electrocatalysts in HER was assessed by linear sweep voltammetry (LSV) with a scan rate of 5 mV/s. The recorded potentials were corrected by considering the corresponding potential drop of electrolyte resistance to monitor only the electrocatalysts' performance. Also, all potentials were converted to the reversible hydrogen electrode (RHE) using the following formula: E(RHE) = E(Ag/AgCl) + 0.199 + 0.059 × pH. The current densities were calculated relative to the geometric surface of electrodes under the electrolyte. The double-layer capacitance (C_dl_) and electrochemical active surface area (ECSA) of electrocatalysts were obtained by conducting cyclic voltammetry (CV) at different scan rates from 10 to 100 mV/s in a potential window around open circuit potential (OCP) (OCP ± 0.1 V). The charge transfer behavior of electrocatalysts was considered by electrochemical impedance spectroscopy (EIS) in the frequency range from 0.1 Hz to 100 kHz. The stability of electrocatalysts was assessed by chronoamperometry at the overpotential in which the current density almost equaled 10 mA/cm^2^.

### Turnover frequency (TOF) calculation procedure

In the HER field, TOF is defined according to Eq. (1)^41^.

1 $$TOF=\frac{number \;of \;produced \;hydrogen \;molecules \;per \;unit \;of \;time}{number \;of \;active \;sites}$$

The number of hydrogen molecules produced per second in HER, when the current density is reported based on the geometric surface of the electrocatalysts, can be calculated from Eq. (2):

2 $$N \left[\frac{molecule}{s}\right]=i \left[\frac{mA}{{cm}^{2}}\right]\times \;geometric \;surface \left[{cm}^{2}\right]\times 3.12\times {10}^{15 }\left[\frac{molecule}{mA s}\right]$$

where N and i are the number of produced hydrogen molecules and current density, respectively, and $3.12\times {10}^{15 }$ molecule/mA s is a conversion factor. The number of active sites can be calculated by considering the electrochemical active surface area (ECSA) and the number of surface atoms per ECSA. To estimate the number of surface atoms per ECSA for Ni_2_P, including both nickel and phosphorous as active sites, the unit cell of Ni_2_P with a volume of 100.54 ${\dot{A}}^{3}$ is taken into account. This unit cell consists of 6 nickel atoms and 3 phosphorous atoms. Therefore, the number of surface atoms per ECSA is calculated by $2.00\times {10}^{15}$ atom/cm^2^. By substituting the obtained terms in formula 1, the TOF is calculated from Eq. 3:

3 $$TOF\left[\frac{molecule}{s atom}\right]=\frac{i \left[\frac{mA}{{cm}^{2}}\right]\times geometric \;surface \left[{cm}^{2}\right]\times 3.12\times {10}^{15 }\left[\frac{molecule}{mA s}\right]}{ECSA [{cm}^{2}]\times 2.00\times {10}^{15}[\frac{atom}{{cm}^{2}}]}$$

## Results and discussion

**Figure 1:**
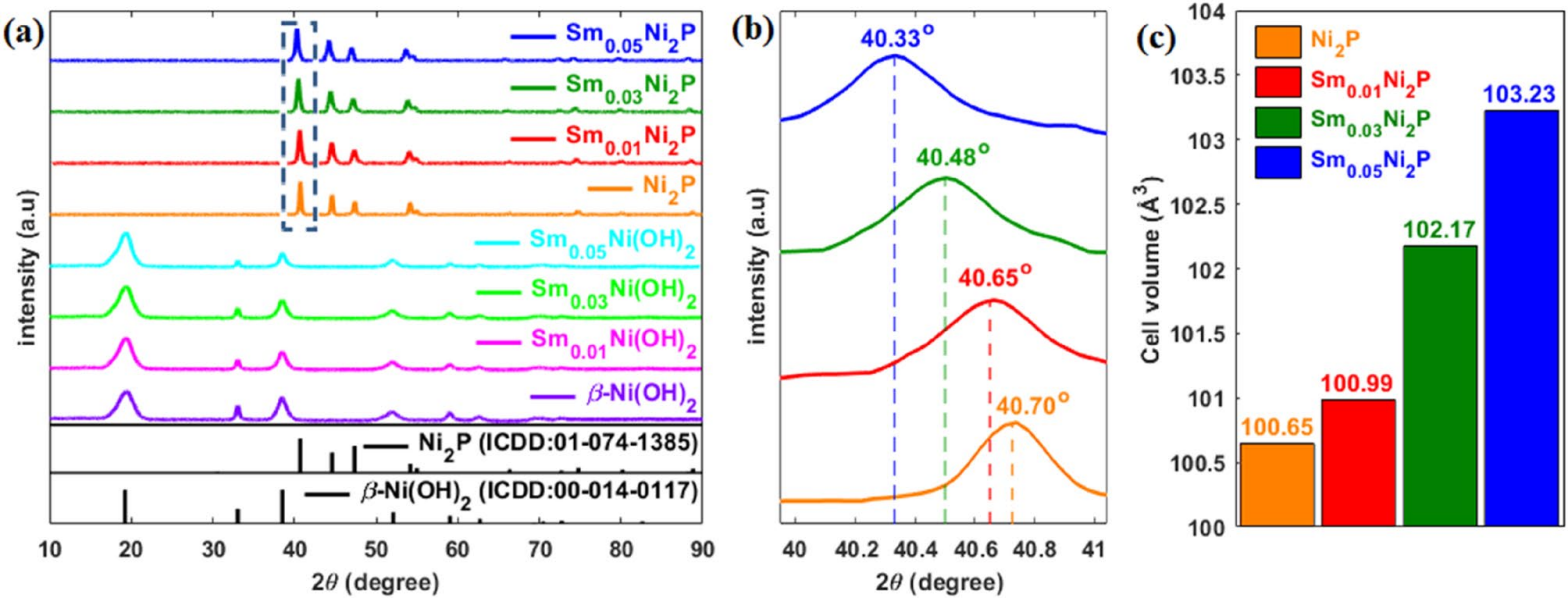
(**a**) XRD pattern of samples; (**b**) high-resolution XRD pattern of nickel phosphide samples around (111) plane of Ni_2_P; (**c**) cell volume of nickel phosphide samples.

The crystalline structure of materials was investigated by XRD test (Fig. 1a). The XRD pattern of pristine nickel hydroxide and Sm-doped nickel hydroxides correspond to the β-Ni(OH)_2_ reference pattern (ICDD: 00-014-0117) without any peak related to Sm-based materials. The XRD pattern of pure and Sm-doped nickel phosphides corresponds well to the Ni_2_P reference pattern (ICDD: 01-074-1385), and no unknown peaks attributed to Sm-containing compounds or impurities are detected. Close examination of XRD patterns reveals that the X-ray diffractogram of Sm-doped nickel phosphides has been negatively shifted to lower angles relative to the pure Ni_2_P pattern. Figure 1b clearly depicts this peak shift around the characteristic pick of Ni_2_P ((111) plane). As per Bragg's law, negative shift in XRD patterns stems from unit cell expansion^42^. Since samarium has a larger atomic radius than nickel, the unit cell expands accordingly when doped to Ni_2_P and replaces the nickel in the structure^43^. This proposition was verified by calculating cell volume for different samples as illustrated in Fig. 1c. Sm-doped Ni_2_P samples have larger cell volume than pure Ni_2_P, and the cell volume increases as the doping level increases, which further confirms successful samarium doping and the proposed explanation for peak shift.

**Figure 2:**
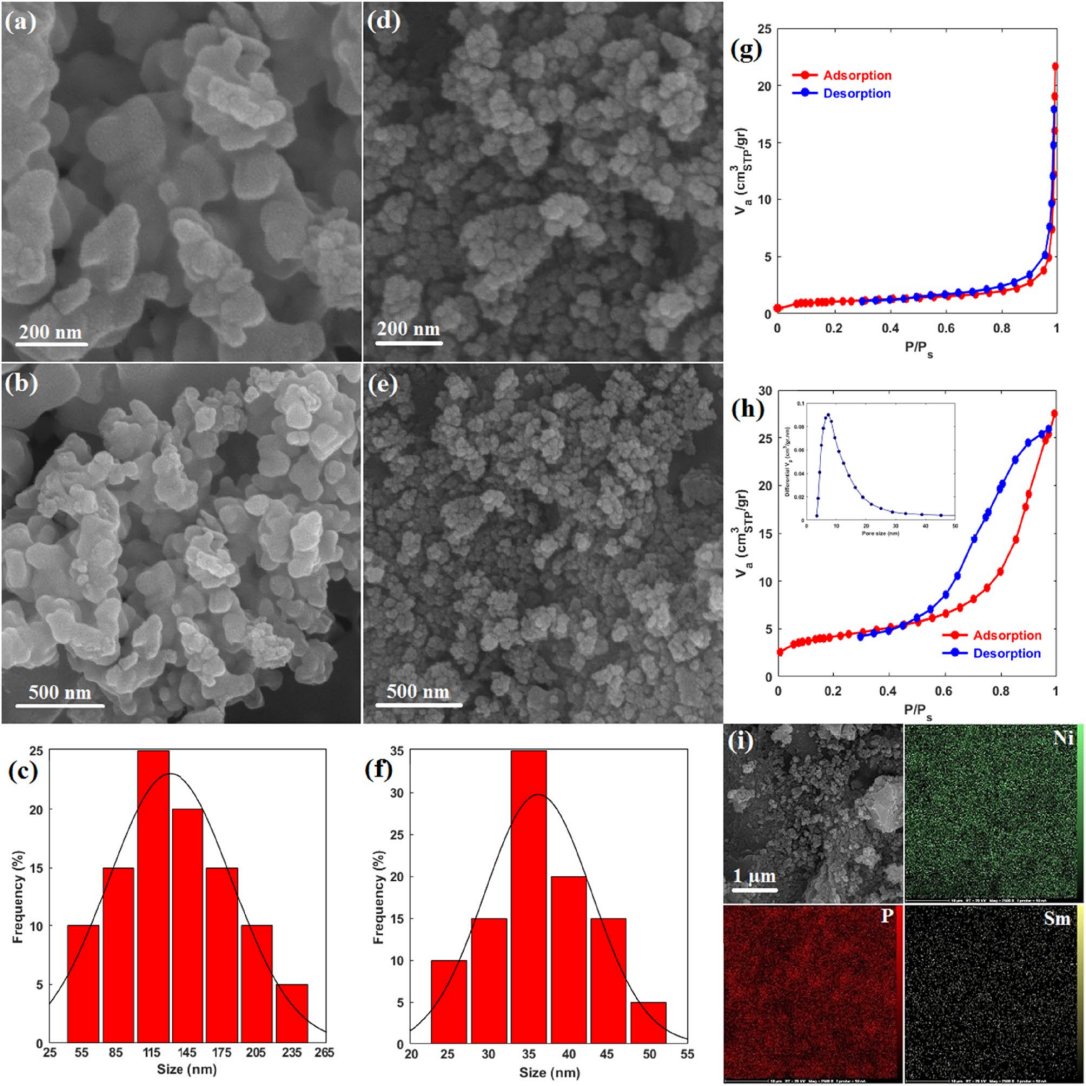
FE-SEM images and the respective particle size distribution of (**a**–**c**) Pure Ni_2_P and (**d**–**f**) Sm_0.03_Ni_2_P; N_2_ adsorption/desorption isotherms of (**g**) Pure Ni_2_P (**h**) Sm_0.03_Ni_2_P; (**i**) SEM image and the respective elemental mapping of Sm_0.03_Ni_2_P.

The morphology and particle size distribution of electrocatalysts were investigated using FE-SEM images. Figure 2a–f depicts FE-SEM images and the corresponding particle size distribution of pure Ni_2_P and Sm_0.03_Ni_2_P. Both pure Ni_2_P and Sm_0.03_Ni_2_P samples exhibit pseudo-spherical agglomerated nanoparticles, and samarium has not noticeably changed the morphology. However, while samarium doping had a negligible effect on the morphology, it considerably affected the particle size. Based on the particle size distributions (Fig. 2c,f), the mean particle size of pure Ni_2_P and Sm_0.03_Ni_2_P was obtained at 136 and 36 nm, respectively, showing that samarium has significantly reduced the particle size. This effect also has been reflected in the SSA of samples. Figure 2g,h show the N_2_ adsorption–desorption isotherms for pure Ni_2_P and Sm_0.03_Ni_2_P, respectively. Based on International Union of Pure and Applied Chemistry (IUPAC) classifications, both isotherms are classified as type IV with a hysteresis of type H1 and H4 for pure Ni_2_P and Sm_0.03_Ni_2_P, respectively, suggesting the samples possess mesoporous structure^44^. The pore size distribution of Sm_0.03_Ni_2_P calculated by the Barrett–Joyner–Halenda (BJH) model with diameters in the range of 2–50 nm clearly confirms the mesoporous structure (inset of Fig. 2h). Accordingly, the Brunauer–Emmett–Teller (BET) model^45^ with an acceptable validity for mesoporous materials was used to calculate SSA. The SSA of pure Ni_2_P and Sm_0.03_Ni_2_P was calculated at 3.77 and 14.76 m^2^/g respectively, which is consistent with the particle size analysis corroborating that samarium doping reduced the particle size. To discover the cause of this effect, the crystallite size of (111) crystalline plane of nickel phosphide samples was calculated using the Scherrer equation (Eq. 5)^46^.

5 $$d=\frac{K\lambda }{WCos\theta }$$

D, K, λ, W, and θ represent average crystallite size, dimensionless shape factor, radiation wavelength (X-ray wavelength), full width at half maximum, and diffraction angle. The crystallite size of (111) plane for pure Ni_2_P, Sm_0.01_Ni_2_P, Sm_0.03_Ni_2_P, and Sm_0.05_Ni_2_P was obtained at 29.01, 28.90, 26.11, and 24.17 nm, respectively, clearly demonstrating that samarium incorporation has reduced the crystallite size. It is because when samarium atoms are doped into the structure of Ni_2_P, they take the place initially held by nickel atoms. Since the atomic radius and valence of samarium and nickel considerably differ, the introduction of samarium makes the structure of Ni_2_P less thermodynamically stable and results in reduced crystallite growth compared to the pure condition^47^. As a result, since crystallites constitute particles, a decrease in crystallite size leads to a corresponding decrease in particle size.

The surface composition and samarium doping amount were determined using EDS (Table 1). The atomic ratio of nickel to phosphorous (Ni:P) in all samples closely aligns with the respective 2:1 stoichiometric ratio in Ni_2_P, corroborating the phase identification by XRD. Also, the atomic ratio of samarium to nickel (Sm:Ni) in the Sm-doped samples acceptably matches the respective nominal ratios, demonstrating precise samarium doping. The surface distribution of elements was studied using elemental mapping (Fig. 2i). The results show the existence of Ni, P, and Sm elements and their uniform distribution across the surface, which verifies successful samarium doping as well as the homogeneity of the sample.

**Table 1:** Surface composition by EDS for different samples.

Sample	Ni (%at)	P (%at)	Sm (%at)	Ni:P	Sm:Ni
Ni_2_P	66.2	33.8	–	1.96	–
Sm_0.01_Ni_2_P	65.3	34.1	0.6	1.91	0.009
Sm_0.03_Ni_2_P	64.4	33.9	1.7	1.9	0.026
Sm_0.05_Ni_2_P	63.2	34.0	2.8	1.86	0.044

**Figure 3:**
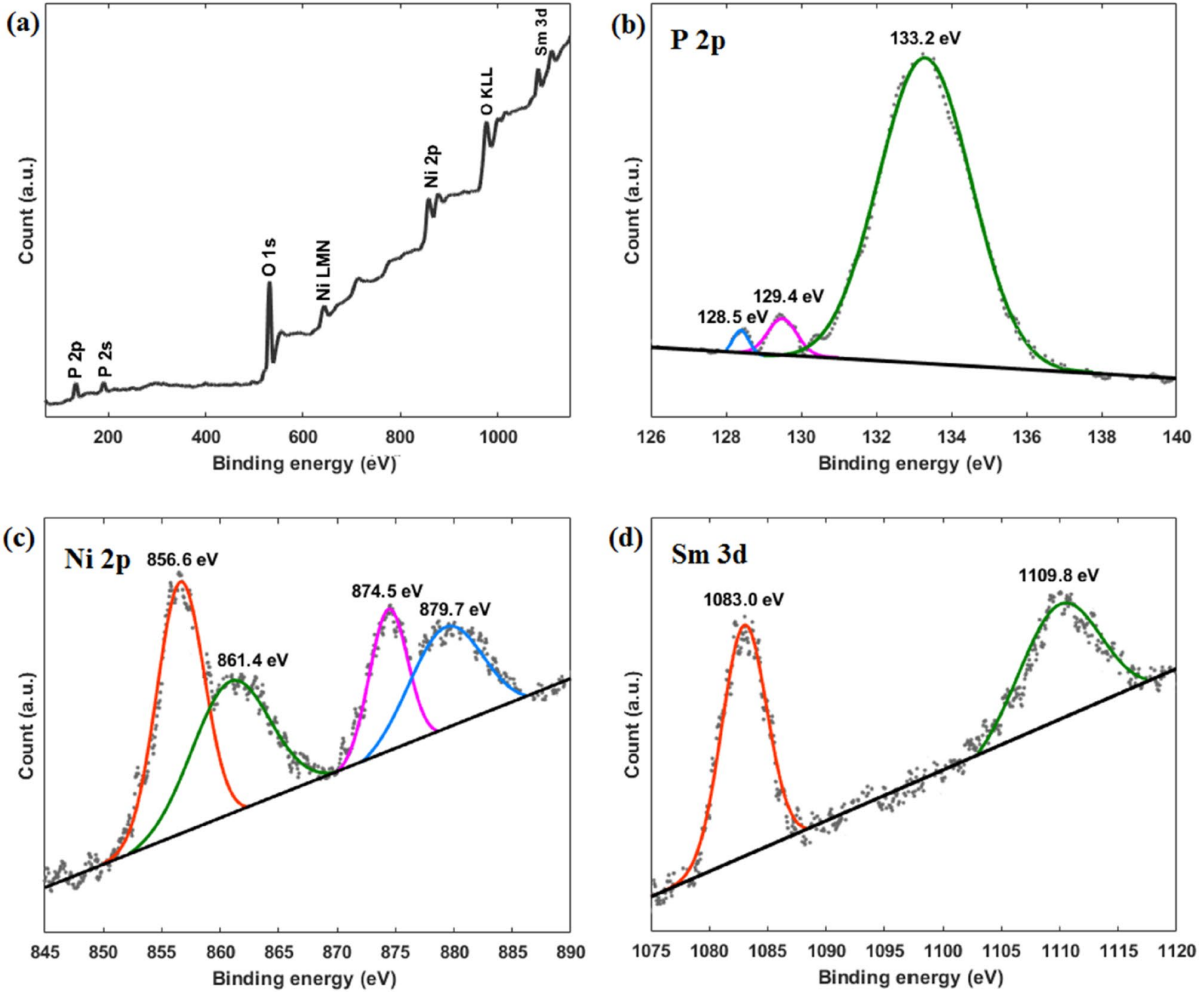
XPS spectrum of Sm_0.03_Ni_2_P in different regions: (**a**) general survey. (**b**) P 2p. (**c**) Ni 2p. (**d**) Sm 3d.

The surface composition of Sm_0.03_Ni_2_P was further analyzed using XPS, as illustrated in Fig. 3. The general survey spectrum (Fig. 3a) reveals distinct peaks related to the energy levels of P, Ni, and Sm elements, indicating their presence on the surface. The high-resolution P 2p energy level spectrum (Fig. 3b) shows two peaks at 129.4 and 128.5 eV, corresponding to P 2p_1/2_ and P 2p_3/2_ energy levels, respectively. Additionally, a peak at 133.2 eV is observed, indicating the presence of oxidized P species.^48^. P 2p energy level peaks have shifted to lower binding energies relative to their normal positions (130.0 eV), indicating that the phosphorous atoms have been partially negatively charged. This shift provides evidence of electron sharing between the nickel and phosphorous atoms, supporting the formation of nickel phosphide compounds^49^. The high-resolution Ni 2p energy level spectrum (Fig. 3c) displays two peaks at 874.5 and 856.6 eV, corresponding to the Ni 2p_1/2_ and Ni 2p_3/2_ energy levels, respectively. Two additional 879.7 eV and 861.4 peaks are assigned to satellites of the corresponding energy levels^50^. The high-resolution Sm 3d energy level spectrum (Fig. 3d) depicts two peaks at 1083.0 and 1109.8 eV, which match Sm 3d_5/3_ and 3d_3/2_ energy levels, respectively. The latter photoelectron lines, which correspond to Sm (III), further confirm the successful doping of samarium^51^.

**Figure 4:**
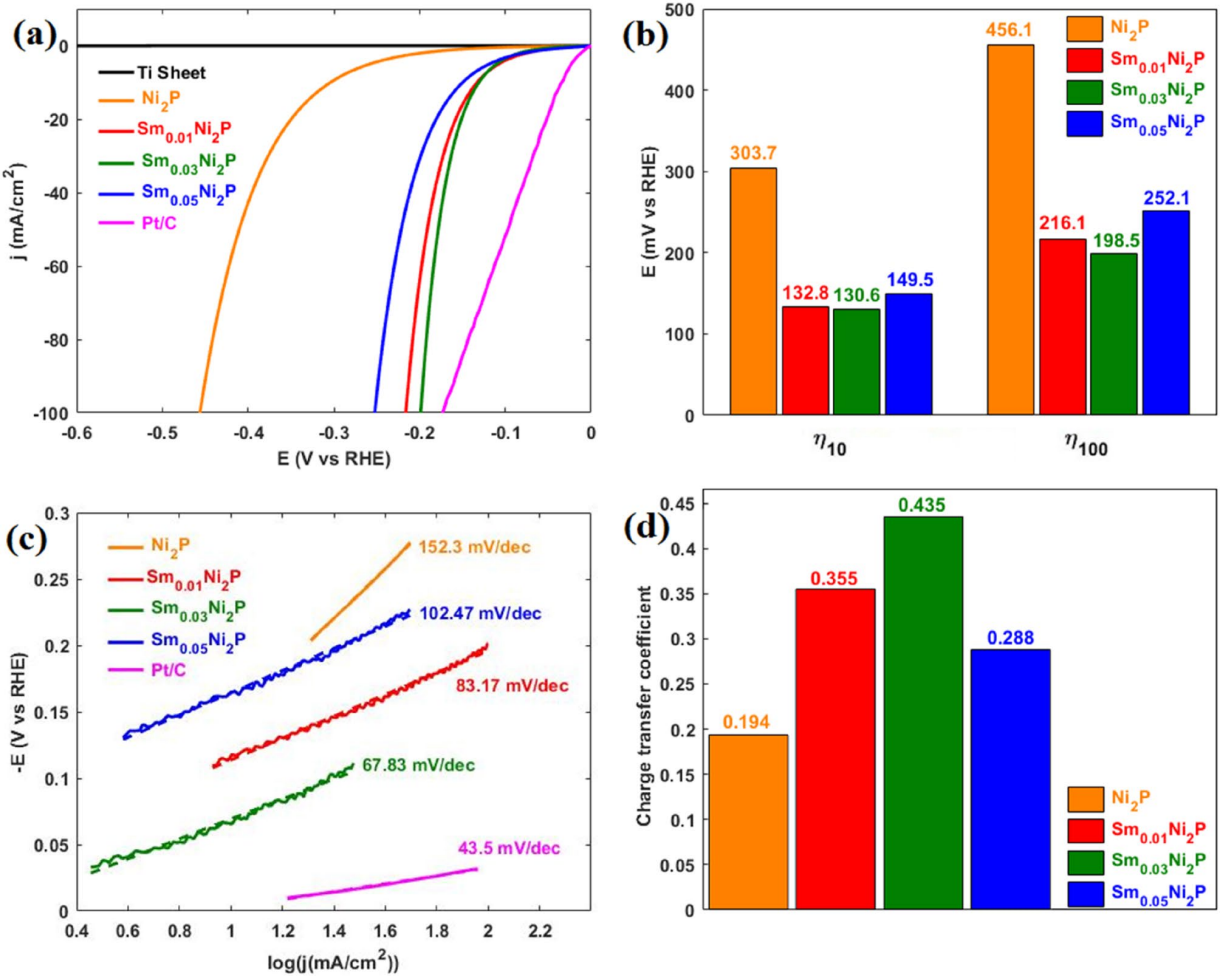
(**a**) LSV curve of electrocatalysts in 1.0 M KOH; (**b**) overpotentials of electrocatalysts at current densities of 10 and 100 $\frac{\text{mA}}{{\text{cm}}^{2}}$; (**c**) corresponding Tafel plot of electrocatalysts; (**d**) charge transfer coefficient of electrocatalysts.

‏The activity of electrocatalysts toward HER was assessed by LSV in a 1.0 M KOH solution at room temperature (Fig. 4a). The bare Ti sheet substrate exhibits negligible activity, indicating that the performance of the electrocatalysts almost entirely stems from the loaded active materials. As observed, samarium can improve the activity of pure Ni_2_P with an optimum doping level of 3%mol. Figure 4b depicts the overpotentials at different current densities for other electrocatalysts. Sm_0.03_Ni_2_P with overpotentials of 130.6 and 198.5 mV at current densities of 10 and 100 mA/cm^2^ shows the highest electrocatalytic activity toward HER and Sm_0.01_Ni_2_P, Sm_0.05_Ni_2_P, and pure Ni_2_P follow in the subsequent rank, respectively.

The kinetics of HER were analyzed using the Tafel plot (Fig. 4c). Ni_2_P with a Tafel slope of 152.3 mV/dec. suggests that it electrocatalyzes the HER via a controlling Volmer reaction, meaning that it suffers from immoderate electrochemical hydrogen adsorption^52^. However, samarium doping has reduced the Tafel slope of pristine Ni_2_P to values that allow the HER to proceed through the Volmer–Heyrovsky mechanism with a fast Volmer reaction and a controlling Heyrovsky reaction, which signifies that samarium doping has moderated the electrochemical hydrogen adsorption^53^. This is most significant at 3%mol of samarium doping, where Sm_0.03_Ni_2_P exhibited the lowest Tafel slope of 67.8 mV/dec., indicating that its highest activity partly originates from its moderate electrochemical hydrogen adsorption. The kinetics of HER was further investigated by analyzing the charge transfer coefficient. The charge transfer coefficient represents the fraction of the interfacial potential used to overcome the energy barriers of an electrochemical reaction and, therefore, can be considered an illustrating indicator of energy efficiency. Figure 4d depicts the charge transfer coefficient of electrocatalysts. Pure Ni_2_P with the lowest charge transfer coefficient demonstrates comparatively poor energy efficiency. However, samarium doping improved the energy efficiency, and Sm-doped Ni_2_P electrocatalysts generally exhibit higher charge transfer coefficients than pure Ni_2_P. Also, Sm_0.03_Ni_2_P possesses the highest charge transfer coefficient, contributing to its better electrocatalytic performance.

**Figure 5:**
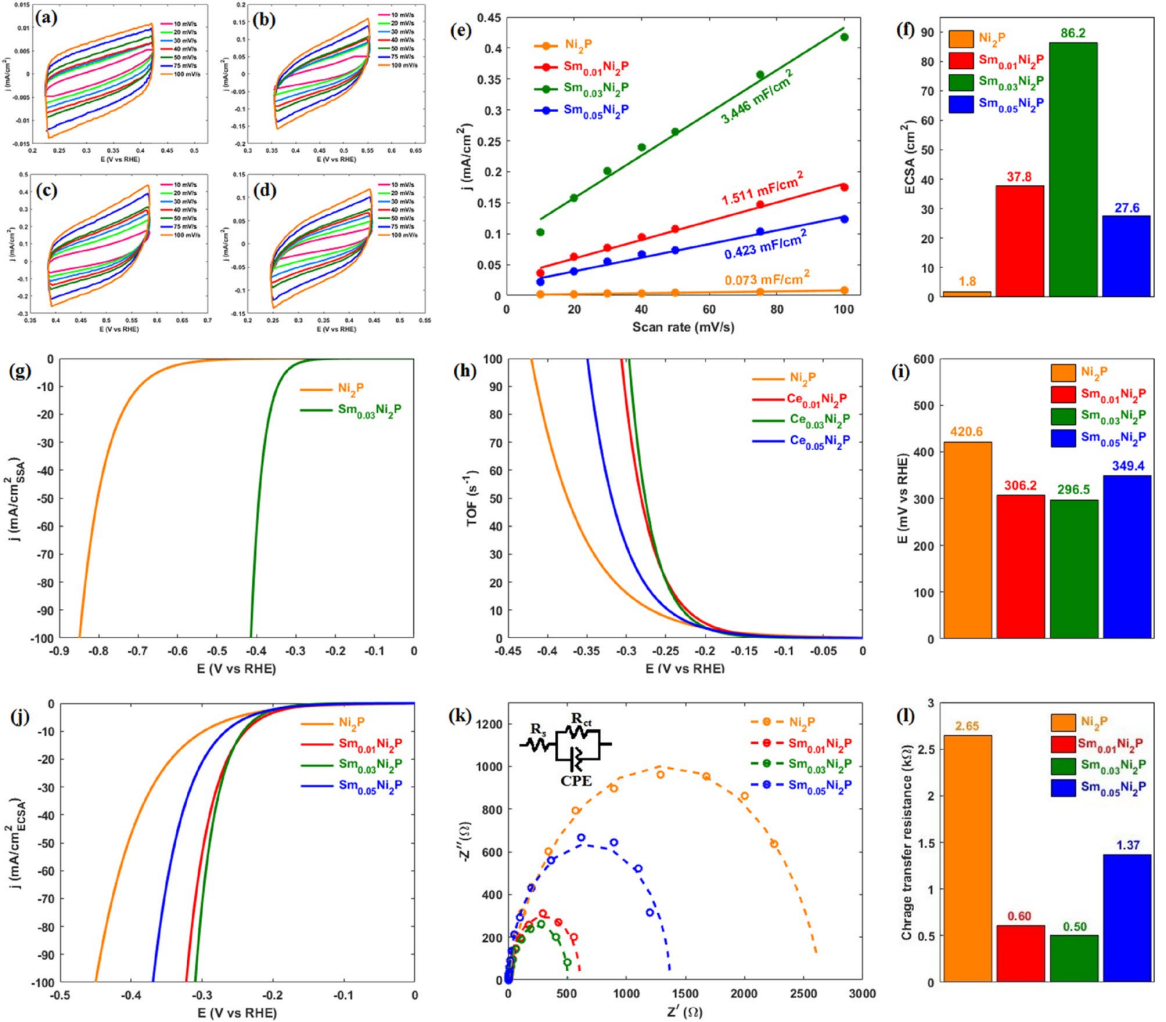
CV curves of electrocatalysts: (**a**) Ni_2_P (**b**) Sm_0.01_Ni_2_P (**c**) Sm_0.03_Ni_2_P (**d**) Sm_0.05_Ni_2_P; (**e**) Capacitive current density vs. scan rate of electrocatalysts; (**f**) ECSA of electrocatalysts; (**g**) LSV curve of Ni_2_P and Sm_0.03_Ni_2_P in 1.0 M KOH normalized to SSA; (**h**) TOF curve of electrocatalysts; (**i**) Overpotential of electrocatalysts at TOF of 100 $\frac{1}{\text{s}}$; (**j**) LSV curve of electrocatalysts in 1.0 M KOH normalized to ECSA; (**k**) Nyquist plot of electrocatalysts; (**l**) charge transfer resistance of electrocatalysts.

The electrochemical active surface area (ECSA) of electrocatalysts was analyzed using the electrochemical double-layer capacitance calculated from the CV test. Figure 5a–d shows the CV curves of different electrocatalysts. The difference between non-faradic currents in anodic and cathodic paths was extracted and plotted against scan rate as shown in Fig. 5e, in which half of the slope indicates the double layer capacitance. As the electrochemical double-layer capacitance and ECSA are linearly proportional to each other, the ECSA was obtained by assuming a specific capacitance of 40 μF/cm^2^ per 1 cm^2^ of ECSA for an electrocatalyst with a smooth surface (Fig. 5f)^54–56^. The surface of pure Ni_2_P exhibits a relatively weak activity toward HER. However, samarium doping has considerably activated the pure Ni_2_P surface. Also, Sm_0.03_Ni_2_P has exposed the largest ECSA, and its highest activity can be related to some extent to its more electrochemically active surface. Given the different SSA of pure Ni_2_P and Sm_0.03_Ni_2_P, to clarify whether the higher activity of Sm_0.03_Ni_2_P solely stems from its larger SSA, the LSV curves were normalized concerning SSA. As shown in Fig. 5g., Sm_0.03_Ni_2_P even after eliminating the effect of SSA demonstrates a higher activity, indicating that samarium doping not only has increased the surface area but also more significantly has activated a previously inactive surface toward HER electrocatalysis.

The effect of samarium doping on the electrocatalytic strength and intrinsic activity of active sites was analyzed using the turnover frequency (TOF) parameter. In the field of HER, TOF is defined as the number of H_2_ molecules an active site generates per unit of time, providing a helpful measure of the electrocatalyst's intrinsic activity. Figure 5h exhibits the TOF curve of different samples. Samarium doping increases the TOF and, consequently, the intrinsic activity of pure Ni_2_P. Namely, Fig. 5i illustrates the needed overpotential of different samples at TOF of 100 $\frac{1}{\text{s}}$, where Sm_0.01_Ni_2_P, Sm_0.03_Ni_2_P, and Sm_0.05_Ni_2_P with overpotentials of 306.2, 296.5, 349.4 mV respectively, require lower overpotential than pure Ni_2_P (420.6 mV) and exhibit greater intrinsic activity. LSV curves normalized with respect to ECSA further illustrate this effect, as Sm-doped electrocatalysts have shown better performance compared to pure Ni_2_P (Fig. 5j). It is worth mentioning that Sm_0.03_Ni_2_P showed the highest TOF, particularly at high overpotentials, and as such, its superior performance could be attributed to its intrinsically more robust active sites.

‏The electrocatalyst-electrolyte interface properties in the electrochemical cell were investigated using electrochemical impedance spectroscopy. Figure 5k exhibits the Nyquist plot of different samples. Pure Ni_2_P, with the largest semi-circle diameter, shows the highest charge transfer resistance. However, samarium doping improved the charge transfer behavior of pure Ni_2_P, as evidenced by the smaller semi-circle diameters and, consequently, lower charge transfer resistances of the Sm-doped Ni_2_P electrocatalysts compared to pure Ni_2_P^57^. The smallest semi-circle diameter was observed for the Sm_0.03_Ni_2_P electrocatalyst, suggesting that its superior activity can be ascribed to a degree to its optimized charge transfer ability. Nyquist plots were further analyzed by defining an appropriate Randles equivalent circuit (see inset of Fig. 5k) for the electrochemical system to provide a quantitative insight into the electrocatalyst-electrolyte interface. Figure 5l illustrates the charge transfer resistance (R_ct_) of electrocatalysts, where consistent with the qualitative analysis, samarium doping decreases the (R_ct_) of pure Ni_2_P by more than 80%, and the lowest charge transfer resistance can be observed by Sm_0.03_Ni_2_P.

**Figure 6:**
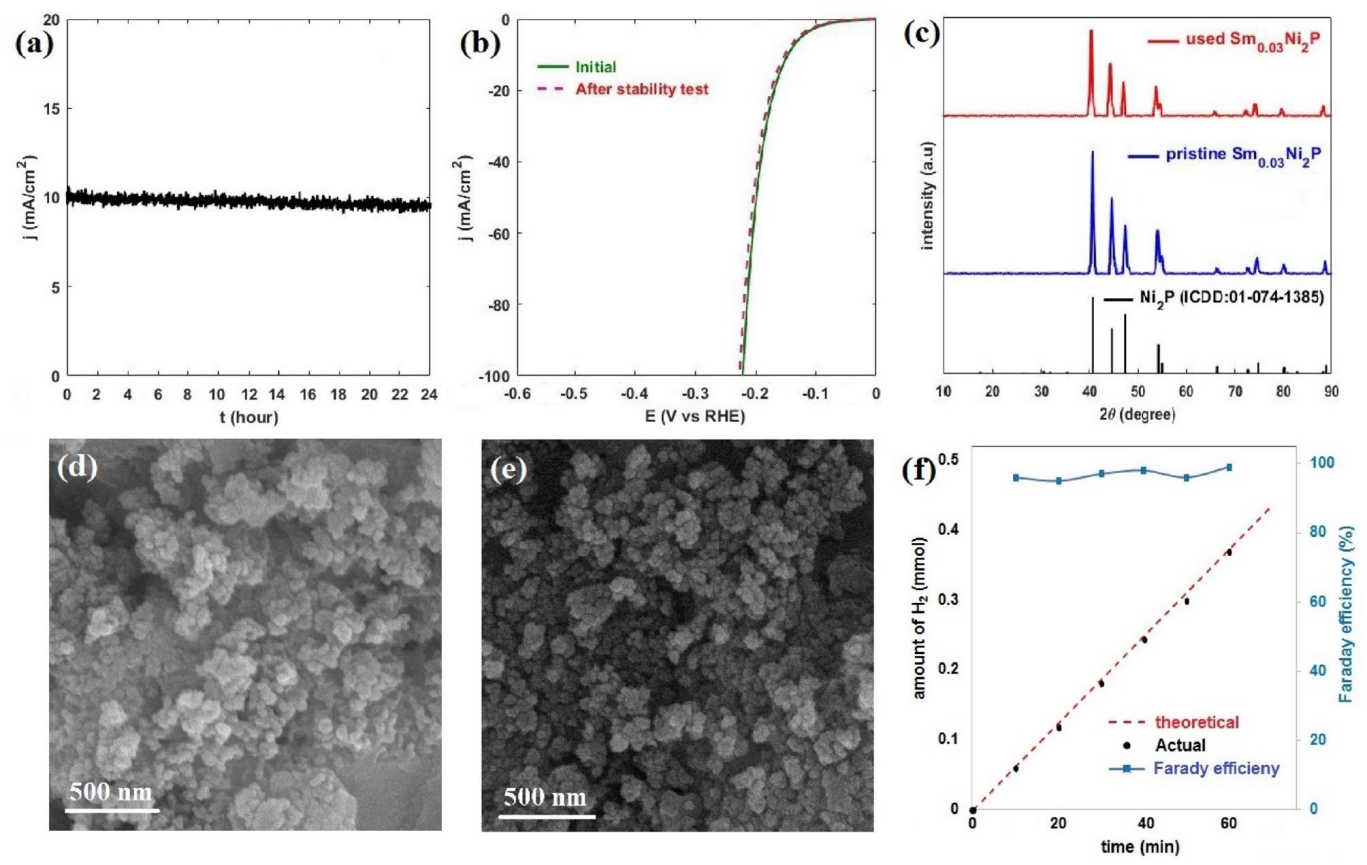
(**a**) Chronoamperometry test of Sm_0.03_Ni_2_P; (**b**) LSV curves of Sm_0.03_Ni_2_P before and after stability test; (**c**) XRD pattern of pristine and used Sm_0.03_Ni_2_P; (**d**) SEM image of used Sm_0.03_Ni_2_P; (**e**) SEM image of fresh Sm_0.03_Ni_2_P; (**f**) Faraday efficiency and amount of actual and theoretical H_2_ evolution of Sm_0.03_Ni_2_P.

The stability and durability of Sm_0.03_Ni_2_P as the best electrocatalyst of the present study were investigated by chronoamperometry to assess its practical application suitability^58^. Figure 6a shows the chronoamperometry for Sm_0.03_Ni_2_P at an overpotential of 0.15 V (vs. RHE), where the steady current density-time plot with less than 5% decrease in activity after 24 h indicates the stable performance of the electrocatalyst. Figure 6b compares the LSV curves of Sm_0.03_Ni_2_P before and after the stability test, where the negligible difference between two activity trends implies the durable performance of the electrocatalyst, suggesting Sm_0.03_Ni_2_P as a high-potential material for real electrochemical applications. The used sample was then characterized after the stability test. Figure 6c compares the XRD patterns of the used electrocatalyst before and after the stability test, revealing no phase change and noticeable alteration in crystalline structure. Figure 6d,e display the SEM images of the used and fresh electrocatalysts, respectively, demonstrating that the morphology and size of the particles have remained almost intact. Consequently, these results further support the notion of the electrocatalyst's stable and durable performance, as well as its robust structure. Finally, the Farady efficiency of Sm_0.03_Ni_2_P was calculated by measuring the amount of evolved H_2_ at a constant current density of 10 mA/cm^2^ at 10-min intervals for 1 h. Figure 6f shows the actual and theoretical amount of hydrogen evolution, where the proximity of these two values leads to Faraday efficiency of nearly 100%, highlighting the exceptional performance of the electrocatalyst.

Sm_0.03_Ni_2_P electrocatalyst, by demonstrating considerable performance in HER, proved to be the best electrocatalyst of this study. Table 2 compares its performance with recent studies that employed transition metal-based materials in alkaline HER. Sm_0.03_Ni_2_P outperformed other electrocatalysts in this study and outclassed multiple other electrocatalysts, highlighting its potential as a promising electrocatalyst for efficient HER.

**Table 2:** Comparison of the present research with recent studies in alkaline HER.

Electrocatalyst	${\eta }_{10}$(mV vs. RHE)	b ($\frac{mV}{dec.}$)	References
Sm_0.03_Ni_2_P	130.6	67.8	Present study
Ce-doped Ni_2_P	158	95.6	^38^
La-doped Ni_2_P	226	126.7	^38^
Ce-doped CoMoP/MoP@C	188	72.2	^59^
Ni_5_P4-NiP_2_-Ni_2_P/NC	202	74	^60^
Ni-doped MoSe_2_	206	81	^61^
Co-doped MoSe_2_	212	99	^61^
Co-doped1T-MoS_2_	240	68	^62^
NiFeP@C	160	78.5	^63^
FeCoP_2_@NPPC	150	79	^64^
NiMoP	400	163	^65^
CoP-CoTe2	178	112	^66^
Ce-doped Ni_2_P	150	76.8	^41^
F-doped carbon dot/CoP	16	89	^67^
N-doped Ni_2_P	110	108	^68^

## Conclusion

In this study, samarium doping was presented as an innovative approach to improve the electrocatalytic performance of Ni_2_P in HER. Samarium doping was carried out at different levels of 1, 3, and 5%mol to comprehensively investigate its effects on the characteristics of pure Ni_2_P. Physical characterization tests demonstrated that samarium doping decreased the particle size of pure Ni_2_P as a result of crystallite growth inhibition and consequently increased the SSA. Electrochemical analyses showed that samarium doping increased the electrocatalytic activity of pure Ni_2_P in HER by modifying the electrochemical hydrogen adsorption, intrinsic activity, SSA, ECSA, and charge transfer behavior. The optimum level of samarium doping was found at 3%mol, where Sm_0.03_Ni_2_P, thanks to its optimized properties, showed a remarkable performance in HER, which introduces samarium and Sm_0.03_Ni_2_P as capable dopant and efficient electrocatalysts for HER, respectively.

## Data Availability

The data that support the findings of this study are available from the corresponding author upon reasonable request.
